# Detection of Depression Severity Using Bengali Social Media Posts on Mental Health: Study Using Natural Language Processing Techniques

**DOI:** 10.2196/36118

**Published:** 2022-09-28

**Authors:** Muhammad Khubayeeb Kabir, Maisha Islam, Anika Nahian Binte Kabir, Adiba Haque, Md Khalilur Rhaman

**Affiliations:** 1 Department of Computer Science Brac University Dhaka Bangladesh; 2 Department of Computer Science and Engineering Brac University Dhaka Bangladesh

**Keywords:** mental health forums, natural language processing, severity, major depressive disorder, deep learning, machine learning, multiclass text classification

## Abstract

**Background:**

There are a myriad of language cues that indicate depression in written texts, and natural language processing (NLP) researchers have proven the ability of machine learning and deep learning approaches to detect these cues. However, to date, these approaches bridging NLP and the domain of mental health for Bengali literature are not comprehensive. The Bengali-speaking population can express emotions in their native language in greater detail.

**Objective:**

Our goal is to detect the severity of depression using Bengali texts by generating a novel Bengali corpus of depressive posts. We collaborated with mental health experts to generate a clinically sound labeling scheme and an annotated corpus to train machine learning and deep learning models.

**Methods:**

We conducted a study using Bengali text-based data from blogs and open source platforms. We constructed a procedure for annotated corpus generation and extraction of textual information from Bengali literature for predictive analysis. We developed our own structured data set and designed a clinically sound labeling scheme with the help of mental health professionals, adhering to the *Diagnostic and Statistical Manual of Mental Disorders*, Fifth Edition (DSM-5) during the process. We used 5 machine learning models for detecting the severity of depression: kernel support vector machine (SVM), random forest, logistic regression K-nearest neighbor (KNN), and complement naive Bayes (NB). For the deep learning approach, we used long short-term memory (LSTM) units and gated recurrent units (GRUs) coupled with convolutional blocks or self-attention layers. Finally, we aimed for enhanced outcomes by using state-of-the-art pretrained language models.

**Results:**

The independent recurrent neural network (RNN) models yielded the highest accuracies and weighted F1 scores. GRUs, in particular, produced 81% accuracy. The hybrid architectures could not surpass the RNNs in terms of performance. Kernel SVM with term frequency–inverse document frequency (TF-IDF) embeddings generated 78% accuracy on test data. We used validation and training loss curves to observe and report the performance of our architectures. Overall, the number of available data remained the limitation of our experiment.

**Conclusions:**

The findings from our experimental setup indicate that machine learning and deep learning models are fairly capable of assessing the severity of mental health issues from texts. For the future, we suggest more research endeavors to increase the volume of Bengali text data, in particular, so that modern architectures reach improved generalization capability.

## Introduction

Major depressive disorder (MDD) is a mental condition characterized by chronic low mood or lack of interest, with a slew of other concerning symptoms over a 2-week period. Depression afflicts an estimated 1 in 15 adults and young adults in a year [1] and is the leading cause of suicide, which is the second-leading cause of mortality worldwide [2,3]. The problem of depression became more pronounced during the COVID-19 lockdown, exacerbating the mental health issues experienced by individuals. It is also a reasonably complex disease to treat because people who suffer from depression are often hesitant to report such symptoms as mental illness remains highly stigmatized in many societies [4]. There exists an abundance of user data or cues related to mental health that can be used by experts to solve such chronic issues.

Data mining in mental health is an advancing field of study that involves the use of machine learning, deep learning, linguistic, and statistical techniques to find patterns in data. Researchers are faced with a range of options with regard to corpus generation from depressive texts: standard academic documents or texts about depression, hashtags, and user posts. Twitter, Facebook, Reddit, and blogs are platforms containing collections of naturally occurring texts. Textual data of internet users depicting symptoms, experiences, thoughts, and conversations about mental health are dispersed across various platforms. In the recent years, it has been observed that individuals, wary of the stigma, prefer to seek clinical help anonymously through writing on platforms, such as Twitter, Reddit, TalkSpace, BeyondMeds, and other social blogs that can connect users with health professionals, counselors, and other users who share similar experiences. eRisk is a specialized platform focused on analyzing early risks using users' texts; Losada et al [5] discussed ways of early detection of depression on the internet. Individuals resort to the option of creating anonymous posts soliciting advice for their conditions in special groups. These posts are often grouped under tags, topics, or even hashtags, such as 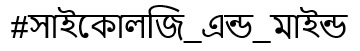 (#psychology_and_mind) and 
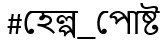
 (#help_post), or in other cases, the social media groups may be dedicated solely to a particular mental health topic.

In psychiatry, symptoms of subjects are generally classified using predefined scales. The Hamilton Depression Rating Scale [6] is an instrument used in scaling depressive disorders. Over the years, various rating scales have emerged that assess symptoms to produce a diagnosis or score. Internet users present various symptoms through texts and often on niche platforms. Extracting these textual data pertaining to mental health and structuring them meaningfully are challenging tasks. A corpus must be generated by domain experts for it to hold validity in the field of psychiatric assessment. For training deep learning architectures, accurately annotated corpora are indispensable.

Ellendorf et al [7] proposed the PsyMine corpus, which was generated by domain experts and their agreement scores were presented. Alonso et al [8] presented a comprehensive review of data mining techniques in the mental health domain. In doing so, the authors covered depression, bipolar disorder, and schizophrenia. Reddit is a standard platform that compartmentalizes mental health posts into subreddits, such as SuicideWatch, bipolar disorder, and anxiety. The Reddit Self-Reported Diagnosis data set is a corpus comprising texts of 9000 reddit users. The corpus was generated with systematic user selection, and the annotation process was crowdsourced [9]. MacAveney et al [10] proposed RSDD-Time, which is a temporal corpus of self-proclaimed diagnosis statements. For each of the statements, the time of diagnosis and whether a condition is present were labeled. Additionally, the authors explored several classification approaches.

The fundamental objectives of this research are to generate an equivalent corpus for the Bengali language and to analyze the data set to detect depression and its severity in individuals. In our research, we integrated natural language processing (NLP) with machine learning and deep learning approaches. Prior work in the field of NLP demonstrated that machine learning and deep learning algorithms are capable of detecting depression-related cues in language [11,12]; however, to date, these efforts have focused on classifying the categories of mental illness rather than their degree. In our research, we adopted unique approaches to detecting and estimating the severity of depression, enabling us to identify those with depression on social media and safeguard others from viewing potentially triggering written content. Furthermore, the prior literature included the identification or categorization of mental diseases from texts in English, German, Russian, and other languages. Bengali is the fourth-most widely spoken language. Hence, we consolidated a process for textual information extraction from Bengali texts and performed lexical and predictive analysis for the purpose of detection of severity of depression.

Several studies in the field of multiclass emotion identification have been conducted using lexicon-based, machine learning, and deep learning approaches. A proposed approach by Mageed and Ungar [13] used gated recurrent neural networks (RNNs) to classify tweets into 24 emotion categories. They yielded over 80% *F*_1_ scores for some categories. Yang et al [14] and Ive et al [15] used a hierarchical architecture with a series of bidirectional encoders to classify different classes of mental health topics. Over the years, focus has shifted toward detection of depression of social media users. Cohan et al [16] created a self-reported depression data set to analyze the language usage of depressed users. They constructed a seed list of keywords assigned to the classes in their data set and applied a Linguistic Inquiry and Word Count (LIWC) approach to compare language usage between a user with and without depression. The experiment also involved the categorization of user posts using logistic regression, extreme gradient boosting (XGBoost), and convolutional neural networks (CNNs) into classes of mental disorders, namely attention deficit hyperactivity disorder (ADHD), bipolar disorder, posttraumatic stress disorder (PTSD), and obsessive-compulsive disorder (OCD). Mustafa et al [17] proposed a novel approach to categorizing depressive texts in English using the LIWC text analysis technique. The posts collected belonged to specific Twitter hashtags, and the authors annotated depressive posts into 3 levels of severity: high, medium, and low. Words associated with mental illnesses were assigned weights, and a support vector machine (SVM) classifier [18], random forest, and 1D CNNs were used in the work. The usage of machine learning and deep learning techniques to scale the level of a condition or situation was proven quite feasible in recent research. For instance, Al‑Garadi et al [19] used transformer models and CNNs to classify mentions of drug usage in English into 4 levels with the help of toxicologists. Identification of depressive texts in the Bengali language has been explored over the recent years through binary text classification techniques. Uddin et al [20] used RNNs to distinguish depressive and nondepressive texts. In the process, they fine-tuned the number of LSTM layers used. Moreover, Khan et al [21] collected Bengali text data from social media and blog posts to assemble a comprehensive Bengali data set containing expressions of positive and negative emotions.

We implemented several baseline models, such as kernel SVMs, complement naive Bayes (NB), logistic regression, random forest, and KNNs. Next, for a deep learning approach, we experimented with convolutional blocks and layers combined with RNNs. Among all the samples classified by bidirectional gated recurrent units (BiGRUs), 81% were correctly identified labels. Bidirectional long short-term memory (BiLSTM) classified 77% of the posts into the correct severity scale. We also reported the results of metrics, such as recall and *F*_1_ scores. In addition, we further explored bidirectional encoder representations from transformers (BERT) models using a pretrained monolingual XLM-RoBERTa language model [22] and expanded on the findings from these techniques.

Various approaches have been followed to identify or categorize depression using English texts, such as multilabel classification of mental disorders and identification of the severity of depression. However, in the context of the Bengali language, only binary classification approaches have been considered and are based solely on the polarity of emotions (ie, happy or sad). This prompted us to conduct research on the identification of hierarchical stages of depressive traits from the literature. For our research, we collected Bengali text data from similar microblogs or accessible social media groups. Generally, social blogs, forums, or groups have mechanisms that classify posts under specific topics pertaining to mental health, and often, these tags are all-encompassing or broad. The social blogs pertaining to mental health would benefit from a hierarchical classification mechanism, where user posts are addressed by professionals or experienced individuals based on urgency. Our technique is unique in the sense that it categorizes a spectrum of negative emotions from a novel Bengali language corpus of self-declared depressive symptoms and emotions. The individual texts were collected from various sources using a web-scraping Application Programming Interface (API) and categorized into 4 levels of severity by experts. Our code has been made publicly available [23].

## Methods

### Study Design

Our approach was twofold. First, we constructed a novel corpus of Bengali texts, consisting of posts exhibiting emotions or symptoms associated with mental illnesses. We studied recommended manuals for the assessment and diagnosis of medical depression to devise a scheme for data annotation. Second, we trained machine learning and deep learning models to classify the Bengali posts according to our scheme.

### Data Set

The Bengali posts were collected from social media platforms and blogs. We used Selenium, a Python web-scraping API, to collect data that originally consisted of code-mixed texts as well as pure Bengali and English texts. Some of the microblogs and social media groups that were relevant to our research included Monojogimon and 
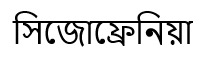
 (schizophrenia) among many others. We automated our program to obtain posts under specific tags or topics. This assisted us in excluding posts that belonged to completely different topics. For blogs and microblogs, the filtering process included separating streams into different topics, such as 
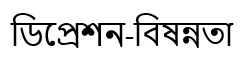
 (depression), 
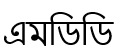
 (MDD), 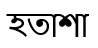 (despair), and others. Among other resources, we collected user posts from Facebook groups, such as 
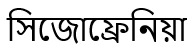
 (schizophrenia) and 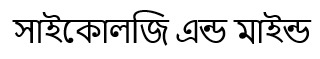 (psychological and mind). Given our research focuses on Bengali literature, we excluded all non-Bengali texts from the data set, which finally contained around 5000 individual posts.

### DSM-5

Reaching an accurate diagnosis is the first step toward appropriately treating any medical condition and mental disorder [24]. The DSM-5 is an authoritative manual that defines and assesses 5 mental disorders. We studied the latest volume of the DSM-5 (2020) to elicit information and improve our understanding of MDD. According to the DSM-5, depression is a condition wherein an individual experiences 5 or more symptoms over the same 2-week period with a pervasive depressed mood or lack of interest and pleasure. The symptoms are a subset of the following:

- Mood swings throughout the majority of the day, practically every day
- Significantly lowered interest in almost all daily activities
- Changes in appetite and notable weight loss or gain
- Diminution in physical movement and a slowing of cognition
- Fatigue or lack of energy nearly every day
- Feelings of worthlessness or excessive and inappropriate guilt on a daily basis
- Reduced ability of concentrating, or indecisiveness, almost every day
- Recurrent thoughts of death, persistent suicidal tendency without a specific plan, or a specific suicide attempt

Furthermore, the DSM-5 underlines the associated features of depression, such as anger, brooding, and compulsive rumination, as well as phobias, excessive concern about physical health, and complaints of pain—all of which were frequently observed in our data set. The handbook discusses how MDD is correlated with comorbidity and mortality, much of which is attributable to suicide. Suicidal ideation manifests actively in those with depression through words such as “I want to kill myself” or passively through remarks such as “I wish I could simply go to sleep and never wake up” [25]. We discovered identical texts in Bengali in our corpus, “
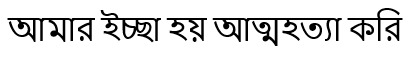
” and “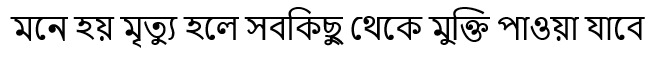,” as instances of suicidal sentiments.

The latest edition of the DSM-5 added 2 specifiers, the presence of manic symptoms and depression with anxiety distress, to further classify diagnoses. This aided in the precision and concentration of our work.

### Labeling Scheme

Upon analyzing the texts in our data set, we found that the linguistic patterns of users with depression in different stages are consistent with the DSM-5 outlines. We devised a comprehensive labeling technique to categorize the texts into 4 distinct classes based on the duration of suffering, the number of symptoms, the use of absolutist words, suicidal ideation, a mention of manic episodes, and delusional thoughts, among other factors. Given that the research focuses on depression and mental health, we opted to consult with mental health specialists to ensure our approach was sound. For verification, we contacted Ms Tasnuva Huque, who is currently a psychosocial counselor at the Counseling Unit of Brac University, Bangladesh. The labeling technique was revised to strictly adhere to DSM-5 criteria, and finally, Ms Huque authenticated the labeling approach for the data set. Level 4 consists of the most acute and concerning cases on our 4-tiered severity scale, with the weight decreasing for subsequent levels up to level 1, which represents the least problematic instances. In the second stage, we were referred to Ms Syeda Tanzila Huque and Ms Ayesha Seddiqa from the Counseling Unit of Brac University.

The involvement of experts from Brac University ensured sound communication and creation of a labeling guideline. The labels were to be assigned with a number of careful considerations. First, the 4 levels of severity were clearly defined and agreed upon by experts. The remaining part of this section outlines the levels in detail.

Level 4 depression is diagnosed when users' texts contain references to past suicidal attempts or suicidal inclinations and thoughts, self-harm as a result of depression, or diagnosis of schizophrenia or borderline personality disorder. Severity level 3 is the broadest category in our data set. It includes texts with references to the need for counseling or medication, postpartum depression or depression during the trimesters, clinical depression associated with psychotic disorders, impaired functioning and phobias (ie, fear of death), lack of appetite, sudden weight loss or gain, delusion, constant mood swings, forgetfulness, breathing difficulties, and other physical health problems. Because this category has a variety of text data and fewer occurrences of each kind, the learning was relatively complex for the models. Severity level 2 consists of written indications of general depression, feelings of hopelessness, loneliness, persistent feelings of instability, and low self-esteem. Lastly, level 1 includes general posts that imply that the users occasionally feel unhappy or that contain mentions of miscellaneous problems that do not pertain to severe depressive symptoms. Some examples of each category are shown in Table 1.

Conflicts during the annotation process arose due to the presence of an array of symptoms belonging to multiple severity levels for a particular item. A user may present hopelessness and elevated levels of frustration along with a statement indicating suicidal ideation. In such cases, the experts resorted to assigning the post the highest level of severity.

Subjective opinion and connotations associated with particular expressions due to their overuse in different contexts presented a problem. Individuals tended to make statements similar to “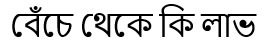,” which translates to “What is the point of living?” Such statements are commonplace and are used to express general frustration, hopelessness, and philosophical contemplation. According to annotation guidelines, such statements were to be handled objectively and not taken as casual statements, since they were extracted from mental health platforms and were written by users with depression. Another general rule prohibited the action of inference from user statements and labels could not be assigned based on inferences. When complex differences of opinion occurred, the posts were marked and the differences were resolved with discussion and majority voting. The label generation process involved physical and online sessions in groups of 2 or 3.

The following section elaborates on the experiments conducted on our data set and explores the best-performing architectures. We tested a total of 5 machine learning models and 11 deep learning models.

**Table 1:** Examples in Bengali and their English translations.

Level and examples (Bengali)		Examples (corresponding English translation)
**Level 4**		
	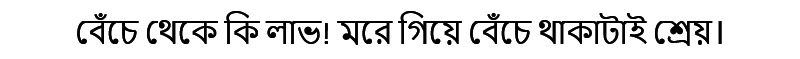	What is the point of living! It is better to die than live.
	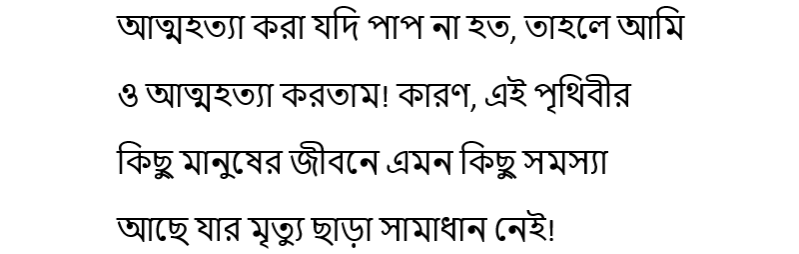	If suicide was not a sin, then I would commit suicide! Because, there are some problems in the life of some people in this world which cannot be solved without death!
	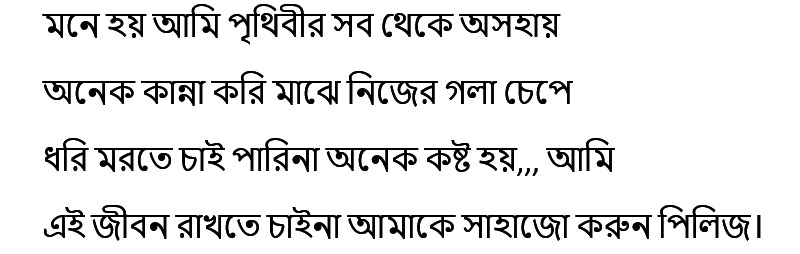	I feel like the most depressed person on this planet and I cry a lot. I want to strangle myself sometimes with my own hand and I want to end my life. But I can’t. It’s very painful. I don’t want to live. Please help me out.
	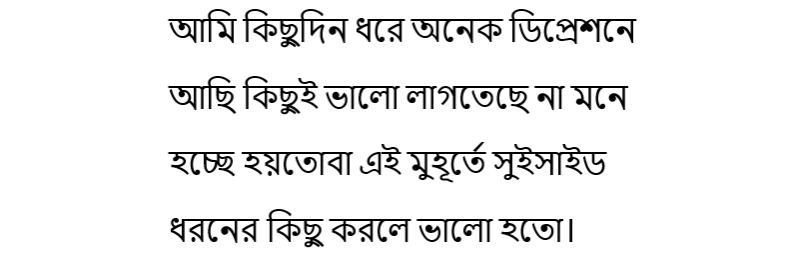	I've been very depressed for a while and nothing seems to be going well, maybe right now if I committed suicide it would be good.
	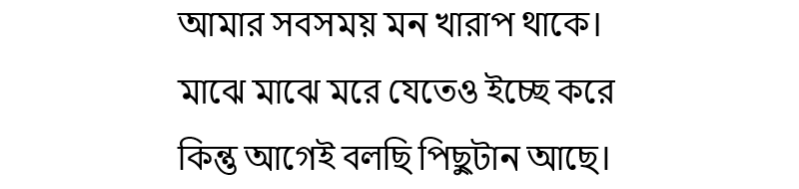	I'm always upset. Sometimes I want to die, but as I said before, there are setbacks.
**Level 3**		
	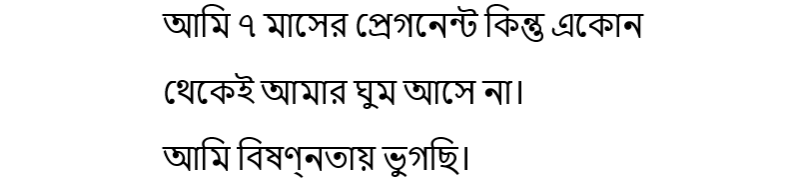	I am 6 months pregnant but I can't sleep. I'm suffering from depression.
	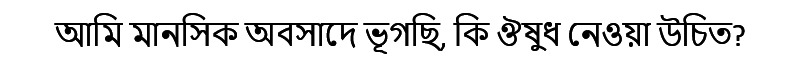	I am suffering from mental depression, what medicine should I take?
	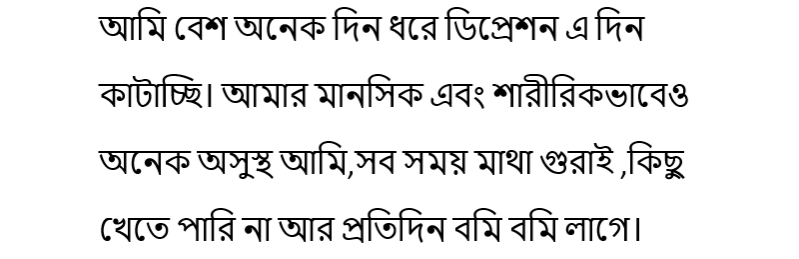	I have been suffering from depression for a long time. I am also very sick mentally and physically, I feel dizzy all the time, I can't eat anything and I feel nauseous every day.
	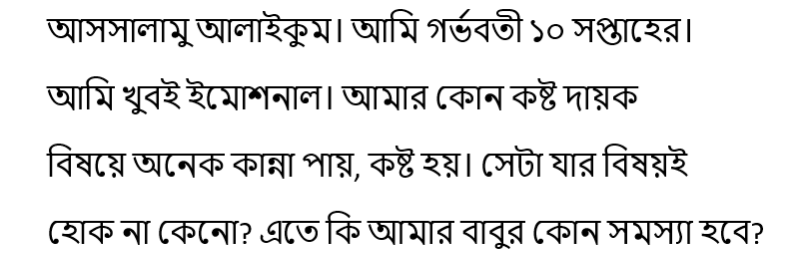	Assalamu Alaikum. I am 10 weeks pregnant. I am very emotional. I cry a lot as a reaction to things that hurt a lot. Whatever the subject may be? Will this be a problem for my baby?
	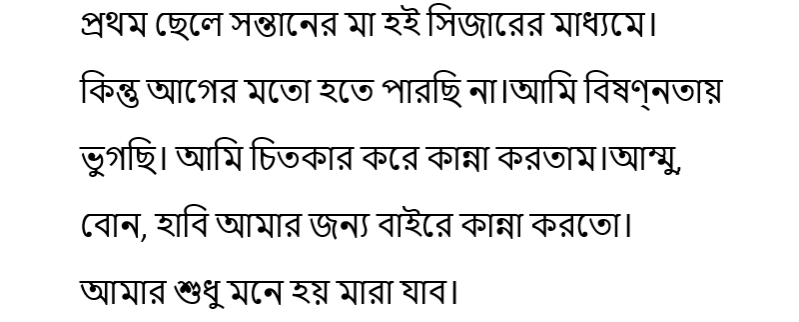	I became the mother of the first child through c-section. But I can't be like before. I am suffering from depression. I used to scream and cry. My mom, sister and my husband used to cry outside for me. I just think I'm going to die.
**Level 2**		
	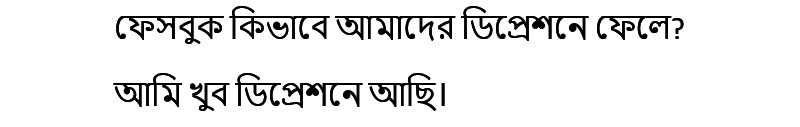	How does Facebook leave us depressed? I'm very depressed.
	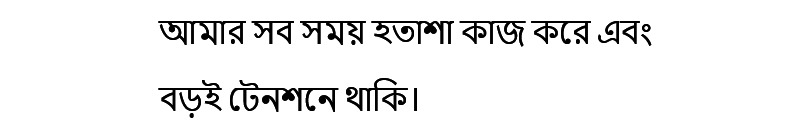	My hopelessness works all the time and I am in great tension.
	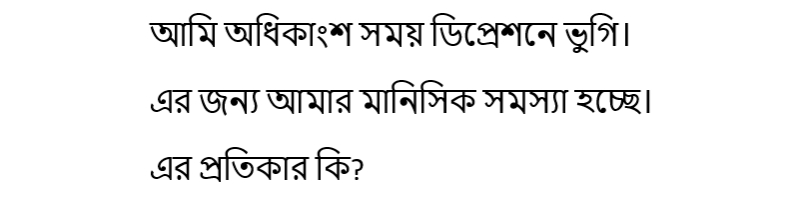	I suffer from depression most of the time. I have mental problems as a result. What is the remedy?
	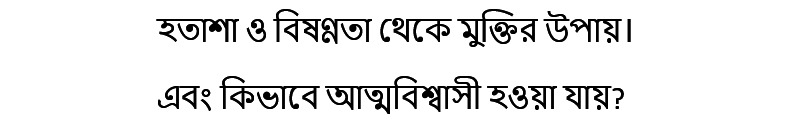	Ways to get rid of frustration and depression. And how to be confident?
	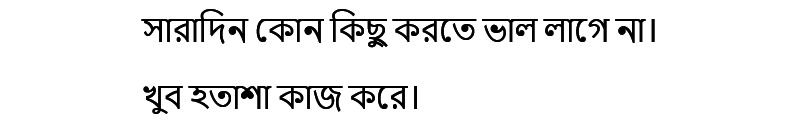	I feel down all day and lack motivation to get things done. I get very depressed.
**Level 1**		
	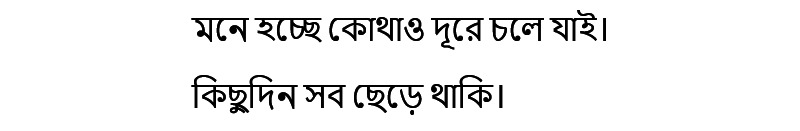	I feel like going somewhere far away. Leaving everything for a few days
	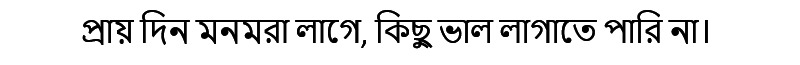	I feel depressed almost every day, don’t feel good about anything
	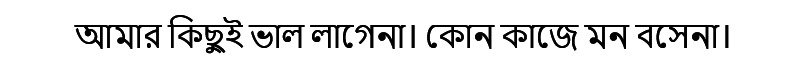	I do not feel well at all. I can’t focus on anything.
	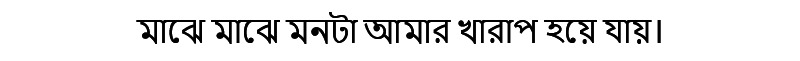	I feel sad sometimes.
	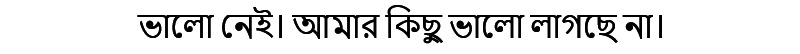	I don't feel well. I don't like anything.

### Preprocessing

The preprocessing stage included the removal of emojis or emoticons, stopwords, numbers, and other foreign characters, followed by tokenization based on whitespace. A pretrained model, FastText [26], was used for the correction [27] of misspelled words. The length of each post varied from 5 to 300 words. To address the imbalanced nature of the data set, class weights were assigned to give more focus to the minority classes.

### Machine Learning Models

Text feature extractions were performed using the bag-of-words (BoW) and term frequency–inverse document frequency (TF-IDF) methods. BoW is a commonly used, simplifying representation of sentences using word frequency. This model disregards grammar or the order of words, while retaining multiplicity. We used BoW to transform texts into feature vectors. TF-IDF is a statistical model that is often used as a weighting factor for information retrieval, text mining, and user modeling. It evaluates how relevant a word is to a post in a collection of documents. A sparse matrix of representation, based on bigram word counts, of the original posts was obtained using this feature extraction scheme.

We applied a set of SVM and complement NB (a member of the NB family) classifiers to the BoW representations [28] and another set of random forest, logistic regression, and KNN classifiers to the sparse matrix from the TF-IDF encoding scheme. We incorporated a grid search to find the best set of hyperparameters for each model (ie, C, gamma, and kernel for the SVM classifier; number of estimators for the random forest classifier; and number of neighbors for the KNN).

### Deep Learning Models

Deep learning architectures have achieved groundbreaking results in multiclass classification. We used some standard deep learning architectures and several variations of 1D convolutional layers incorporated with neural network classifiers.

Word embeddings are vector representations of words used as underlying input representations. They generally enhance the performance of sentiment analysis tasks to a great extent. FastText provides word embeddings for 157 languages, including Bengali. Some of the additional benefits of FastText include its extension of the Skip-gram algorithm from Word2vec to create character-level representation of words. In our research, we chose FastText word embedding for the embedding layers. Young et al [29] explored the deep learning trends in text classification. The following section describes how we extracted lower-level sequences from texts and captured long-range dependencies. It also discusses standard deep learning architectures that were used in our experiments.

**Figure 1:**
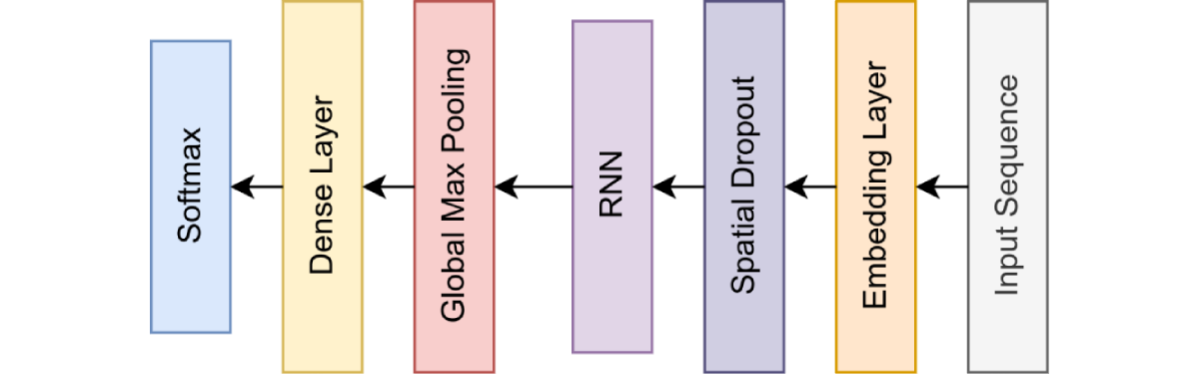
A recurrent neural network (RNN).

**Figure 2:**
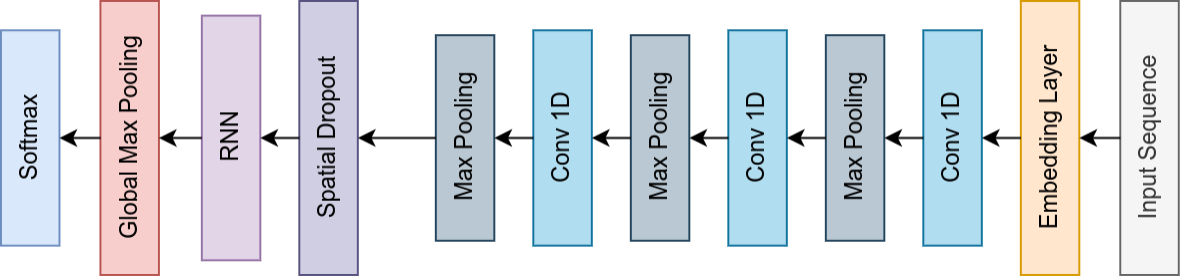
RNN with convolutional blocks. RNN: recurrent neural network.

Figures 1 and 2 show some of the generalized architectures used in our experiments. The experimental design used 1 or a combination of these architectures.

#### BiLSTM

A BiLSTM connects 2 layers from opposite directions, which enables the architecture to propagate past or future information in both directions. The introduction of LSTM can be traced back to work by Hochreiter and Schmidhuber [30]. The forget, input, and output gates work to capture dependencies and update the contemporary memory cell. The following equations denote the operation of a unidirectional LSTM architecture:

i_t_ = σ(W_i_ × [h_t–1_, x_t_] + b_i_)

f_t_ = σ(W_f_ × [h_t–1_, x_t_] + b_f_)

q_t_ = tanh(W_q_ × [h_t–1_, x_t_] + b_q_)

o_t_ = σ(Wo × [h_t–1_, x_t_] + bo)

c_t_ = f_t_ ⊙ c_t–1_ + i_t_ ⊙ q_t_

h_t_ = o_t_ ⊙ tanh(c_t_)

The inputs at the current time, the forget gate, and the output gate are represented by x_t_, f_t_, and o_t_, respectively. The outputs from these gates update the memory cell c_t_ and the current hidden state h_t_. The sigmoid function denoted by σ has its domain in the range (0,1). The hyperbolic tangent function has outputs lying between (–1,1). The f_t_ function controls how much information is to be retained, and the input gate stores relevant information.

#### BiLSTM and Self-Attention

The Self-Attention layer takes sequences as inputs and outputs aggregate attention scores to find out on which sequences to focus. In our approach, the bidirectional layers were followed by the Self-Attention and GlobalMaxPooling layers. Bahdanau et al [31] proposed state-of-the-art attention architecture that generates context vectors by taking weighted summations of the input vectors and of the hidden cells. Equal emphasis is put on all words of the input sentence, unlike in traditional BiLSTM. In the following equations, the context vector is denoted by c_i_ and α_ij_ refers to the weights that are calculated through backpropagation. h_j_ refers to the j-th word in the input sequence.

C_i_ = Σα_ij_h_j_

The weighted sums were calculated for t_x_ annotations. Moreover, the weights α_ij_ were computed using the softmax function. Several researchers have applied attention mechanisms for text classification and reported that the results have exceeded those of simpler architectures [32,33].

#### Deep CNN

CNNs use the concept of sliding a kernel across a tensor to create feature maps. These feature maps capture the important features throughout the text to gain some understanding about the text. The sliding kernel operation on a feature vector over a single channel can be summarized using the following equation:

c = f(w^T^ × x_i:I h+1_ + b)

Conneau et al [34] explored deep CNNs and concluded that performance increases with depth. Their architecture comprised convolutional blocks, each convolutional block having 2 convolutional layers along with a batch normalization layer and ReLU nonlinearity. The fully connected layers come after the K-Max Pooling layer. Our architecture comprised 3 1D convolutional layers with 3×3 kernel sizes and 512 dense layer units. The 3 pooling layers used had pool sizes of 3, 5, and 14, respectively. The model was trained with 20 epochs and with a batch size of 32.

#### Deep CNN-BiLSTM

Hassan and Mahmood [35] proposed a convolutional recurrent architecture for sentence classification. They modified the standard CNN-LSTM architecture by excluding the pooling layers. In our experiment, we modified the standard architecture by placing the pooling layers after each of the convolutional layers, as shown in Figure 2. Many authors have compared the CNN-LSTM architecture with stand-alone CNNs, LSTM-CNNs, and other variations [36,37]. In our case, we placed the deep convolutional blocks with the pooling layers. Next, the pooled output, which had a minimal dimension, was passed to an LSTM that learned the ordering of the local features that were extracted.

Experiments were conducted further by modifying the aforementioned architectures with the addition of Self-Attention layers or by changing encoders. The experimental models included a deep CNN-BiGRU, a deep CNN-BiLSTM with Self-Attention, a deep CNN-BiGRU and Self-Attention, and a deep CNN-Self-Attention.

#### GRUs

This architecture excludes the output gate and has fewer parameters. It consists of 2 gates, a reset gate and an update gate. The reset gate deals with the short-term memory of the architecture.

r_t_ = σ(x_t_ × U_r_ + H_t–1_ × W_r_)

u_t_ = σ(x_t_ × U_u_ + H_t–1_ × W_u_)

The first step of this model involves the computation of candidate hidden states, which is determined by the hidden state of the previous timestamps and multiplied by the reset gate output. The resulting output from the tanh activation function is the candidate hidden state.

Ĥ_t_ = tanh(x_t_ × U_g_ + (r_t_ ᵒ H_t–1_) × W_g_

The extent of information a candidate gate can harbor is determined by the reset gate. The candidate hidden state is then used to calculate the current hidden state. GRUs alone or when used as part of other hybrid architectures have proven to be successful [38,39].

#### Pretrained Language Models

The Hugging Face Transformer library offers a variety of pretrained language models [40]. Devlin et al [41] proposed a novel language representation model known as BERT. The model is trained on vast text data to learn bidirectional representations, and the architecture provides room for task-specific fine-tuning. The first part of training involved the implementation of a masked language model. A small proportion of the words were replaced with a fixed token to mask them. The model was trained to predict the masked tokens based on the context. To make the BERT model suitable for classification, a classification token was inserted at the start of the first sentence and a separator token was placed at the end. Additionally, the tokens were assigned a sentence and positional embeddings. A classification layer was placed after the transformer model for emotion detection or sentiment analysis tasks.

#### XLM-RoBERTa

This model develops on BERT and uses richer vocabulary for pretraining on multilingual corpuses [42,43]. The XLM-RoBERTa architecture used in our experiments comprised a pretrained model trained on an ~3 GB monolingual Bengali corpus [22].

### Evaluations

For our imbalanced classification problem, we used class-weighted evaluation metrics, such as the weighted *F*_1_ score, weighted precision, and recall. For the weighted *F*_1_ score, we adjusted the *F*_1_ scores of each class according to the proportion of samples in that class. The macro-*F*_1_ score returned the average *F*_1_ score without considering the number of samples for each class label. Thus, it was insensitive to class imbalance.

*F*_1_ score = 2 × (Precision × Recall)/(Precision + Recall)

Weighted *F*_1_ score = [(N_1_ × Class_1__
*_F_*_1_) + (N_2_ × Class_2__
*_F_*_1_) + (N_3_ × Class_3__
*_F_*_1_) + (N_4_ × Class_4__
*_F_*_1_)]/(N_1_ + N_2_ + N_3_ + N_4_)

## Results

### Traditional Models

We compared the real labels of pure Bengali texts with model predictions and summarized the traditional machine learning models (see Tables 2 and 3). The SVM with a linear kernel achieved the highest generalization ability on TF-IDF vector representations with 78% accuracy. Moreover, this result was marginally better than the SVM model that was trained on the same representations with the radial basis function (rbf) kernel, meaning that the representations are linearly separable to some extent. The following are the values for the hyperparameters that were obtained via a grid search:

BoW:

- kernel: rbf
- C: 55
- gamma: 0.008

TF-IDF:

- kernel: linear
- C: 1

Moreover, the random forest model, which was trained using 25,000 estimators with class weights assigned accordingly to account for the class imbalance, achieved an accuracy of 75%.

**Table 2:** Results with BoW^a^ embedding.

Model	Precision	Recall	*F*_1_ score	Accuracy
Kernel SVM^b^-rbf^c^	0.73	0.74	0.73	0.74
Kernel SVM-linear	0.71	0.72	0.71	0.72
Complement NB^d^	0.66	0.66	0.66	0.66

^a^BoW: bag-of-words.

^b^SVM: support vector machine.

^c^rbf: radial basis function.

^d^NB: naive Bayes.

**Table 3:** Results with TF-IDF^a^ vectorizer.

Model	Precision	Recall	*F*_1_ score	Accuracy
Kernel SVM^b^-rbf^c^	0.76	0.77	0.76	0.77
Kernel SVM-linear	0.77	0.78	0.76	0.78
Random forest	0.76	0.75	0.72	0.75
Logistic regression	0.74	0.74	0.74	0.74
KNN^d^	0.70	0.53	0.44	0.53

^a^TF-IDF: term frequency–inverse document frequency.

^b^SVM: support vector machine.

^c^rbf: radial basis function.

^d^KNN: K-nearest neighbor.

### Deep Learning Architectures

We also reported the weighted *F*_1_ scores, macroaverage *F*_1_ scores, and accuracies of our deep learning architectures for pure Bengali texts. Our recurrent models had the following setup: embedding layers, followed by 1D spatial dropout, a stack of recurrent units, and dense layers. The 1D spatial dropout drops the entire feature map; in other words, it drops a feature along with its correlated neighbors by setting its activations to 0. The spatial dropout rate varied from 0.1 to 0.4.

Furthermore, for feature extraction using CNNs, the deep convolutional block was placed after the embedding layer, followed by the 1D spatial dropout. All the models were trained with batch sizes of 32 and 64. The LSTM unit achieved the highest weighted *F*_1_ score and an accuracy of 0.78. The following Table 4 details the results of our final deep learning architectures. The BiGRU reached a weighted *F*_1_ score of 0.81, while the additional layers were able to distinguish the classes moderately well. The BERT architecture was adjusted with a batch size of 8, a learning rate of 1 × 10^–4^, and a fully connected layer consisting of 4096 units with L1 and L2 regularizers set to 0.01. The model was trained for 400 epochs on an NVIDIA RTX 3060 GPU.

Table 5 details the performance of the BiGRU on individual severity levels. It was able to distinguish severity level 4 posts with 81% accuracy. Moreover, severity level 1 and 2 posts could be detected with 86% and 82% accuracy, respectively. It is also important to note that the CNN-based recurrent models achieved higher accuracies in the case of level 4 severity detection. The deep CNN-BiGRU, in particular, achieved 83% accuracy and the deep CNN-BiLSTM yielded 82% accuracy.

The objective of our research was to maximize recall as an indicator since a false-negative case might create a hindrance for a suicidal individual in getting help. The BiGRU achieved a precision of 88%, so it might filter out most of the severe cases if the metric were to be maximized.

**Table 4:** Results of deep learning implementations.

Model	Precision	Recall	Accuracy	*F*_1_ score	*F*_1_ score (macroaverage)
BiGRU^a^	0.81	0.81	0.81	0.81	0.78
BiLSTM^b^ Self-Attention	0.73	0.72	0.72	0.73	0.70
Deep CNN^c^-BiLSTM	0.80	0.77	0.77	0.78	0.76
Deep CNN-BiLSTM Self- Attention	0.77	0.76	0.76	0.76	0.74
BiLSTM	0.77	0.77	0.77	0.77	0.74
BiGRU Self-Attention	0.75	0.74	0.74	0.74	0.73
Deep CNN-BiGRU	0.76	0.76	0.76	0.76	0.74
Deep CNN-BiGRU Self- Attention	0.75	0.73	0.73	0.74	0.73
Deep CNN Self-Attention	0.77	0.77	0.77	0.77	0.75
Monolingual XLM-RoBERTa-BiGRU^d^	0.78	0.78	0.78	0.78	0.75

^a^BiGRU: bidirectional gated recurrent unit.

^b^BiLSTM: bidirectional long short-term memory.

^c^CNN: convolutional neural network.

^d^BERT: bidirectional encoder representations from transformers.

**Table 5:** BiGRU^a^ implementation breakdown for each label.

Scale	Precision	Recall	Accuracy	*F*_1_ score
Severity level 1	0.85	0.86	0.86	0.86
Severity level 2	0.78	0.82	0.82	0.80
Severity level 3	0.63	0.62	0.62	0.63
Severity level 4	0.88	0.80	0.80	0.84

^a^BiGRU: bidirectional gated recurrent unit.

## Discussion

### Principal Findings

This paper discussed an empirical study that identified the severity of depression using Bengali text-based data. Before categorizing, different cases from classes 1 through 4 were thoroughly studied. The findings suggest that by combining machine learning and deep learning approaches, substantial accuracy may be attained for linguistics data sets on complex psychological tasks, such as the analysis of depression.

### Analysis

In our context, the performance of stand-alone RNNs exceeded expectations due to several reasons. First, composite models, such as BERT, tend to produce average results with small multilabel data sets [44]. Second, the order of words in our data set was significant in concluding the nature of a person’s mental state. The stand-alone RNN model captures short-to-medium-range dependencies from input sentences. For example, in the consecutive sentences extracted from the data set “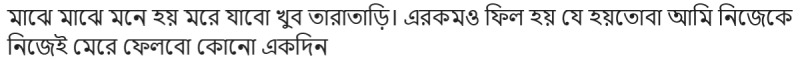,” the anonymous individual writes, “I will die,” or “
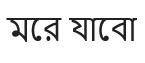
.” In the following sentence, they are no longer passive about the issue and state, “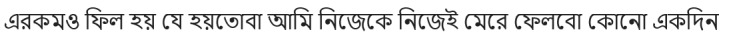,” or “I feel like I will probably kill myself someday,” indicating a possible suicide attempt in the future. Convolutional and pooling layers tend to disrupt information about the local order of words that must be captured for proper classification. Lastly, in the majority of the cases, our labeling criteria put emphasis on absolutist words, such as “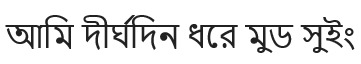 (mood swing), 
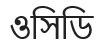
 (OCD), 
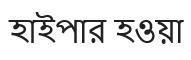
 (hyper) 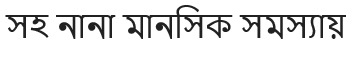 (mental issues)’ and “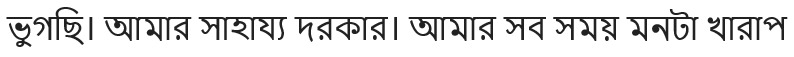 (feel upset all the time) 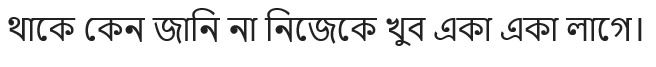.” The attention model, which assigns attention weights to input representations, requires a substantially larger corpus to accurately calculate which word token is to be assigned a higher weight.

Previous studies performing linguistic analysis on depressive English texts have focused heavily on identification of specific emotions or mental health issues. In doing so, the authors have performed multilabel classification on a corpus collected from Reddit. Research incorporating Bengali text data is largely limited to classification of depressive and nondepressive texts only. Therefore, the significance of this research lies in demonstrating that deep learning classifiers not only identify specific emotions or conditions but also classify the level of severity. Second, it continues research on Bengali NLP to include classification of hierarchical depressive labels.

The evidence collected from the studies demonstrates that sequential deep learning architectures produce quality results. A proportion of the hybrid architectures suffered from limitations due to unavailability of Bengali language resources. Many state-of-the-art techniques benefit from an abundance of textual data belonging to an array of social topics. However, for categorizing niche social topics in low-resource languages, RNN models generalize better without requiring a large corpus and high computational power.

### Limitations

The lack of pure Bengali texts was a constraint to our work. Bengali-speaking people write texts in Romanized Bengali, which is the representation of Bengali language in English scripts. We were unable to use a fraction of the data initially collected, because some of the texts were code-mixed or written in Romanized Bengali. Moreover, Bengali is a low-resource language and the user posts in our corpus belonged to specialized social topics.

### Conclusion

Future research might focus on designing experiments using Romanized Bengali texts, too. Despite the limitations of the study, our models' overall performance and findings indicate that machine learning and deep learning models are reasonably robust and suitable to identify the severity of mental health conditions.

## Abbreviations

API: Application Programming Interface
BERT: bidirectional encoder representations from transformers
BiGRU: bidirectional gated recurrent unit
BiLSTM: bidirectional long short-term memory
BoW: bag-of-words
CNN: convolutional neural network
KNN: K-nearest neighbor
LIWC: Linguistic Inquiry and Word Count
LSTM: long short-term memory
MDD: major depressive disorder
NB: naive Bayes
NLP: natural language processing
OCD: obsessive-compulsive disorder
rbf: radial basis function
RNN: recurrent neural network
SVM: support vector machine
TF-IDF: term frequency–inverse document frequency

## References

[1] Torres F What Is Depression?.

[2] Bachmann S (2018). Epidemiology of suicide and the psychiatric perspective. Int J Environ Res Public Health.

[3] World Health Organization Depression.

[4] Barney LJ, Griffiths KM, Jorm AF, Christensen H (2006). Stigma about depression and its impact on help-seeking intentions. Aust N Z J Psychiatry.

[5] Losada DE, Crestani F, Parapar J (2020). eRisk 2020: self-harm and depression challenges. Advances in Information Retrieval. ECIR 2020. Lecture Notes in Computer Science, Vol 12036.

[6] Hamilton M (1960). A rating scale for depression. J Neurol Neurosurg Psychiatry.

[7] Ellendorff T, Foster S, Rinaldi F (2016). The PsyMine Corpus - a corpus annotated with psychiatric disorders and their etiological factors.

[8] Alonso SG, de la Torre-Díez I, Hamrioui S, López-Coronado M, Barreno DC, Nozaleda LM, Franco M (2018). Data mining algorithms and techniques in mental health: a systematic review. J Med Syst.

[9] Yates A, Cohan A, Goharian N (2017). Depression and self-harm risk assessment in online forums. Proceedings of the 2017 Conference on Empirical Methods in Natural Language Processing.

[10] MacAvaney S, Desmet B, Cohan A, Soldaini L, Yates A, Zirikly A, Goharian N RSDD-Time: temporal annotation of self-reported mental health diagnoses. arXiv..

[11] Medhat W, Hassan A, Korashy H (2014). Sentiment analysis algorithms and applications: a survey. Ain Shams Eng J.

[12] Gkotsis G, Oellrich A, Velupillai S, Liakata M, Hubbard TJP, Dobson RJB, Dutta R (2017). Characterisation of mental health conditions in social media using informed deep learning. Sci Rep.

[13] Mageed M, Ungar L (2017). EmoNet: fine-grained emotion detection with gated recurrent neural networks.

[14] Yang Z, Yang D, Dyer C, He X, Smola A, Hovy E (2016). Hierarchical attention networks for document classification.

[15] Ive J, Gkotsis G, Dutta R, Stewart R, Velupillai S (2018). Hierarchical neural model with attention mechanisms for the classification of social media text related to mental health.

[16] Cohan A, Desmet B, Yates A, Soldaini L, MacAvaney S, Goharian N SMHD: a large-scale resource for exploring online language usage for multiple mental health conditions. arXiv..

[17] Mustafa R, Ashraf N, Ahmed FS, Ferzund J, Shahzad B, Gelbukh A, Latifi S (2020). A multiclass depression detection in social media based on sentiment analysis. 17th International Conference on Information Technology–New Generations (ITNG 2020). Advances in Intelligent Systems and Computing, Vol 1134.

[18] Boser BE, Guyon IM, Vapnik VN (1992). A training algorithm for optimal margin classifiers.

[19] Al-Garadi M, Yang Y-C, Cai H, Ruan Y, O'Connor K, Graciela G-H, Perrone J, Sarker A (2021). Text classification models for the automatic detection of nonmedical prescription medication use from social media. BMC Med Inform Decis Mak.

[20] Uddin AH, Bapery D, Arif ASM (2019). Depression analysis from social media data in bangla language using long short term memory (LSTM) recurrent neural network technique.

[21] Khan MRH, Afroz US, Masum AKM, Abujar S, Hossain SA, Hassanien AE, Bhattacharyya S, Chakrabati S, Bhattacharya A, Dutta S (2021). A deep learning approach to detect depression from Bengali text. Emerging Technologies in Data Mining and Information Security. Advances in Intelligent Systems and Computing, Vol 1300.

[22] Hugging Face Indic-Transformers Bengali XLMRoBERTa.

[23] omanwhatiscomputer / depression-severity.

[24] Rachel AD (2014). DSM-5 handbook of differential diagnosis. Am J Psychiatry.

[25] Truschel J Depression Definition and DSM-5 Diagnostic Criteria.

[26] Grave E, Bojanowski P, Gupta P, Joulin A, Mikolov T (2018). Learning word vectors for 157 languages.

[27] Kaiser A Bengali Word Spelling Correction Using Pre-trained Word2Vec.

[28] Zhang Y, Jin R, Zhou Z (2010). Understanding bag-of-words model: a statistical framework. Int J Mach Learn Cyber.

[29] Young T, Hazarika D, Poria S, Cambria E Recent trends in deep learning based natural language processing. arXiv..

[30] Hochreiter S, Schmidhuber J (1997). Long short-term memory. Neural Comput.

[31] Bahdanau D, Cho K, Bengio Y (2015). Neural machine translation by jointly learning to align and translate.

[32] Jing R (2019). A self-attention based LSTM network for text classification. J Phys: Conf Ser.

[33] Li W, Qi F, Tang M, Yu Z (2020). Bidirectional LSTM with self-attention mechanism and multi-channel features for sentiment classification. Neurocomputing.

[34] Conneau A, Schwenk H, Barrault L, Lecun Y (2017). Very deep convolutional networks for text classification.

[35] Hassan A, Mahmood A (2018). Convolutional recurrent deep learning model for sentence classification. IEEE Access.

[36] Sosa PM (2017). Twitter Sentiment Analysis using combined LSTM-CNN Models.

[37] Zhou C, Sun C, Liu Z, Lau F A C-LSTM neural network for text classification. arXiv..

[38] Yan W, Zhou L, Qian Z, Xiao L, Zhu H (2021). Sentiment analysis of student texts using the CNN-BiGRU-AT model. Sci Prog.

[39] Zhou L, Bian X (2019). Improved text sentiment classification method based on BiGRU-Attention. J Phys: Conf Ser.

[40] Wolf T, Debut L, Sanh V, Chaumond J, Delangue C, Moi A, Cistac P, Rault T, Loaf R, Fuctowicz M, Davison J, Shleifer S, Platen P, Ma C, Jernite Y, Plu J, Xu C, Scao TL, Gugger S, Drame M, Lhoest Q, Rush A (2020). Transformers: state-of-the-art natural language processing.

[41] Devlin J, Chang M, Lee K, Toutanova K BERT: pre-training of deep bidirectional transformers for language understanding. arXiv..

[42] Liu Y, Ott M, Goyal N, Du J, Joshi M, Chen S, Levy O, Lewis M, Zettlemoyer L, Stoyanov V RoBERTa: a robustly optimized BERT pretraining approach. arXiv..

[43] Conneau A, Khandelwal K, Goyal N, Chaudhary V, Wenzek G, Guzm F, Grave E, Ott M, Zettlemoyer L, Stoyanov V Unsupervised cross-lingual representation learning at scale. arXiv..

[44] Ezen-Can A A comparison of LSTM and BERT for small corpus. arXiv..

